# Potential Global Invasion Risk of Scale Insect Pests Based on a Self-Organizing Map

**DOI:** 10.3390/insects14070572

**Published:** 2023-06-21

**Authors:** Jun Deng, Junjie Li, Xinrui Zhang, Lingda Zeng, Yanqing Guo, Xu Wang, Zijing Chen, Jiali Zhou, Xiaolei Huang

**Affiliations:** State Key Laboratory of Ecological Pest Control for Fujian and Taiwan Crops, College of Plant Protection, Fujian Agriculture and Forestry University, Fuzhou 350002, China; 1150203008@fafu.edu.cn (J.L.); xinruiz0387@163.com (X.Z.); lingdazeng@126.com (L.Z.); anoka@foxmail.com (Y.G.); gkcjs500@foxmail.com (X.W.); yanguan1210@outlook.com (Z.C.); julyzzhou@126.com (J.Z.)

**Keywords:** scale insects, self-organizing maps, global establishment risk, invasive species

## Abstract

**Simple Summary:**

The self-organizing map (SOM), an unsupervised artificial neural network model, has emerged as a tool for analyzing insect species assemblages associated with geographic regions. By comparing regions that share similar pest assemblages, SOM provides available information to rank potential invasive species. Scale insects, an important group of Hemiptera, attract a great deal of attention from researchers and quarantine authorities due to their significant threats to crops and ornamental plants. There are few studies on distribution pattern and predicting the invasion of scale insects at a global scale. In the present study, a global presence⁄absence dataset, including 2486 scale insect species in 157 countries, was extracted to assess the establishment risk of potential invasive species based on a self-organizing map (SOM). We provide preliminary establishment risk assessments of numerous scale insects at a global scale and confirm that SOM can be a reliable tool for analyzing a large number of species simultaneously.

**Abstract:**

In the present study, a global presence/absence dataset including 2486 scale insect species in 157 countries was extracted to assess the establishment risk of potential invasive species based on a self-organizing map (SOM). According to the similarities in species assemblages, a risk list of scale insects for each country was generated. Meanwhile, all countries in the dataset were divided into five clusters, each of which has high similarities of species assemblages. For those countries in the same neuron of the SOM output, they may pose the greatest threats to each other as the sources of potential invasive scale insect species, and therefore, require more attention from quarantine departments. In addition, normalized ζ_i_ values were used to measure the uncertainty of the SOM output. In total, 9 out of 63 neurons obtained high uncertainty with very low species counts, indicating that more investigation of scale insects should be undertaken in some parts of Africa, Asia and Northern Europe.

## 1. Introduction

With economic globalization, increasing global tourism and trade leads to increasing numbers of insect pest invasions into non-native areas. Invasive pests introduced into new areas may undergo an explosive increase in numbers and rapid spread, particularly when natural enemies are absent [1]. In the USA, about 40% of insect pests are considered to be invasive species, which cause at least USD 13 billion in crop losses each year [2,3]. To reduce the possibility of alien species invasions, it is necessary to identify those species most likely to invade and which locations are most vulnerable at the global scale, which is also the basis for determining appropriate management strategies such as field control, phytosanitary measures or standardizing industry practices on the survival of the pest [1,4,5]. Some bioclimatic methods including GARP [6], MaxEnt [7] and CLIMEX [8] have emerged to predict the potential distribution of invasive species. However, these methods require accurate location information of observed occurrences and related environmental variables as inputs, which is unsuitable for country-level occurrence data. In addition, most studies using these methods usually only estimate the potential distribution or risk of a few species [5].

The self-organizing map (SOM) [9,10], an unsupervised artificial neural network model, focuses on pattern recognition, clustering and the visualization of multidimensional data [1], and has emerged as a tool for analyzing insect species assemblages associated with geographic regions [4,11]. The SOM performs significant data reduction from high-dimensional data into two-dimensional space that can be usefully interpreted [1,11]. The SOM hypothesizes that the pest assemblage of a region is a result of all complex interactions of biotic and abiotic factors [1,11,12]. By comparing regions that share similar pest assemblages, the SOM provides available information to rank potential invasive species. It should be pointed out that the result score of the SOM is not considered as a probability of establishment, although the score looks like a certain probability between 0 (species absence) and 1 (species presence). An establishment probability of invasive pests should include a time frame related to several processes (arrival, survival, establishment and spread). The term “relative establishment risk score” is used in the present study to help us gain a better understanding of the result scores of the SOM.

The SOM has been confirmed as a robust method for ranking potential invasive species from a large dataset and making an overall assessment of invasion risk [1,13,14,15,16]. The advantage of using the SOM is that it can analyze hundreds or thousands of presence/absence species data from different regions simultaneously. For example, Worner and Gevrey identified potentially high-risk invasive pest species of 844 mainly phytophagous insect pests from 459 geographical areas [1]. Morin et al., tried to assess global weed assemblages and likelihoods of 6690 plant species becoming weeds using the SOM analysis [17]. Qin et al., used the SOM to estimate the global establishment risk of 180 economically significant fruit fly species distributed in 118 countries [5].

Scale insects (Hemiptera: Coccoidea), an economically important plant-feeding group, contain about 8000 described species in the world [18]. Many scale insects are pests, especially those on fruit trees, woody ornamentals and shrubs. This group includes some notorious pests such as *Ceroplastes rubens*, *C. rusci* and *Coccus hesperidum* [19,20]. The economic loss caused by scale insects in the USA has been estimated to exceed USD 500 million each year [21]. Meanwhile, some scale insects have a strong adaptability to new environments and are capable of causing serious economic damage when they colonize a new region. Of the 255 introduced species in the USA, about 75% are considered serious pests [22]. In the early 1970s, the introduction of the cassava mealybug, *Phenacoccus manihoti*, from South America to West Africa caused considerable damage to cassava throughout Africa and threatened the major staple of 200 million people [23]. Diagnostics of scale insects is difficult and of limited use for non-experts, especially with imperfect specimens [24]. As a consequence, it is necessary to make appropriate management strategies ahead of time for quarantine so as to assist in preventing invasions of scale insects into non-infested regions.

To assess the global establishment risk of scale insects and identify potentially invasive scale insect species, we implemented the SOM method to determine geographic patterns and species assemblages of scale insects. The worldwide distribution data of scale insects were collected and used to: (1) generate risk lists of scale insects for different countries; (2) cluster the countries with the most similar species assemblages; and (3) identify the potential distribution of some possible invasive scale insects for China.

## 2. Materials and Methods

Data were initially extracted from the ScaleNet database [18]. The database covered most areas in the world and included different types of information on the distribution of scale insects, representing various countries, states or provinces. To assess the invasive risk of the scale insect species for each country, country-level presence/absence data were used in this study. A total of 7905 scale insects within 157 countries were included in an initial dataset. Many scale insects were never reported in other countries after they were described as a new species in one place. For the SOM method based on the similarity of pest species assemblages, species restricted to only one country cannot provide information. Thus, we excluded such species from the SOM analysis. The final dataset was a 157 × 2486 matrix comprising 157 vectors (countries) each with 2486 elements (species), where each element represented the presence (1) or absence (0) of species in a country. The original data file is available in Table S1.

Each cell/neuron of the output layer using the SOM has a virtual vector that could be interpreted as a virtual pest species assemblage (the range from 0 to 1), indicating the possibility that a species will occur in that cell [1]. If one species is absent in a country, the value represents the risk index of invasion of the species. According to Vesanto’s rule [25], the neuron/cell number of the SOM map could be initially determined as the formula m = 5√n, where n represented the number of vectors (157 countries). The topographic and quantification errors (TE and QE) were used to estimate SOM quality [10]. The final number of neurons is set as close to Vesanto’s heuristic rule as possible (m = 62). For this study, the map resolution of 7 × 9 was set as the most appropriate size for further analysis. The ‘batch’ training was used for the algorithm with a gaussian neighborhood function and 500 × 63 = 31,500 iterations as training length, and the TE and QE were 0.0127 and 6.85, respectively. After the training of data was finished, each country was assigned to a best matching unit (BMU), and species assemblages were the most similar in the same BMU (the same weight vectors). While each neuron of the SOM map represented a cluster, it was of interest to define larger clusters by regrouping the adjacent neurons that were expected to have similar weight vectors to each other [11]. Larger clusters contributed to a better understanding of the clustering pattern of pests at a global scale. However, it was difficult to directly detect the boundaries of larger clusters on the trained SOM map. To define the cluster boundaries of the SOM map, a hierarchical cluster analysis with a Ward linkage method [26] was applied [1,11,27]. The Davies–Bouldin index (DBI) [28] was then calculated to select the number of clusters with the minimum DBI value.

To assess the cluster validity of the results, ζ diversity was applied to measure the uncertainty of the SOM output [12]. ζ_i_ diversity was defined as the number of species shared between any i sites in the same neuron, as a measure of compositional similarity [12,29]. The normalized ζ_i_ (ζ_i_/ζ_1_) was used as a clustering validity measure. Three different situations of assemblage similarity based on the ζ diversity measure were shown as follows: (1) if a very low relative ζ_2_ value compared to the other neurons, the uncertainty of assemblage similarity was supposed to be high; (2) a high ζ_2_ value with low groupwise similarity ζ_3–5_ was considered as a medium uncertainty; while (3) high ζ_2_ and high ζ_3–5_ simultaneously indicated a low uncertainty.

The SOM analysis was performed using the SOM toolbox (version 2.0 beta) [25] under a Matlab environment (Matlab R2016b, The Mathworks, Inc., Natick, MA, USA). The SOMVIS Package [30], an add-on for the Matlab SOMToolbox, was also applied to define cluster boundaries with Ward’s linkage method. All maps included in this study were generated using ArcGIS 9.2 (ESRI, Redlands, CA, USA). The assessment of the uncertainty of SOM was performed in the packages zetadiv [31] available in R 3.3.1 [32].

## 3. Results

**Figure 1:**
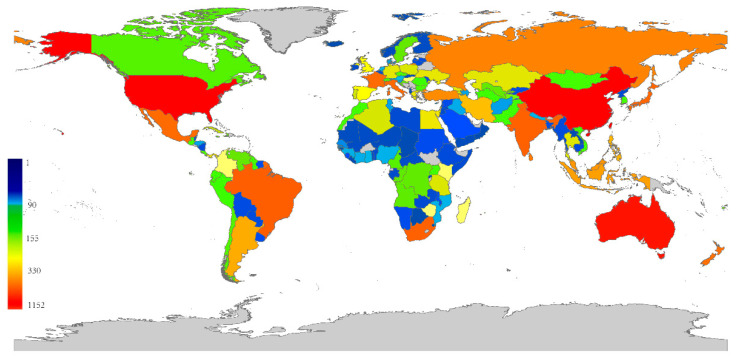
The distribution map of scale insects. Number of scale insects in each country is indicated by color density.

The distribution map of scale insects based on distribution data of 7905 species across 157 countries is shown in Figure 1. For each continent, the numbers of scale insect species ranked from high to low are as follows: Asia (3060), Africa (1608), North America (1532), Oceania (1224), Europe (1064) and South America (999). The top eight countries by species number were China (1152), USA (1049), Australia (819), Mexico (590), Brazil (526), India (512), South Africa (508) and Japan (491). Though Africa ranked in second place with 1608 scale insect species in total, 71% of African countries had less than 100 species.

**Figure 2:**
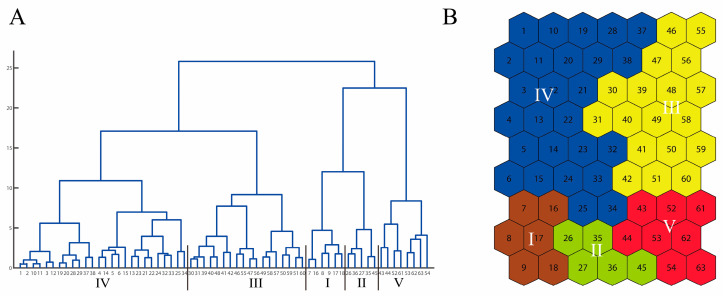
(**A**) Dendrogram of the cluster analysis; (**B**) the five clusters defined on the self-organizing map using a Ward linkage method.

**Figure 3:**
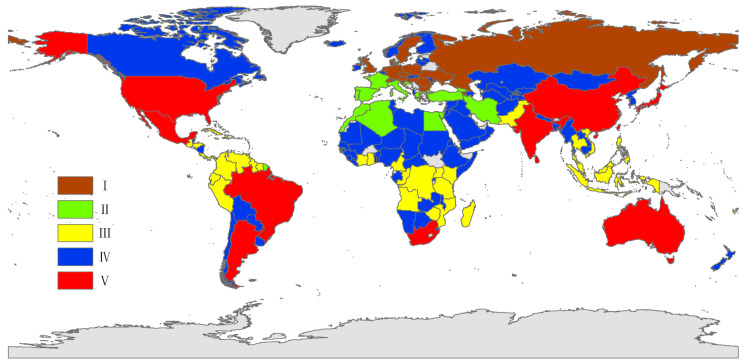
The five clusters including the 157 countries represented by different colors.

Based on the dendrogram (Figure 2A), all countries included in our study were divided into five clusters with the lowest DBI value (0.87), as shown in Figure 2B. Each cluster was plotted on the world map using different colors (Figure 3). Most countries clustered together on the SOM map were geographically close. The countries from cluster I represented most countries in northern Europe. Some Mediterranean countries including Greece, Israel, Spain, etc., constituted cluster II. Cluster III included larger tropical areas, such as some countries in South America and the central region of Africa and Southeast Asia. The countries of Cluster IV were very scattered including some countries in South America (Chile, Paraguay, Bolivia and Uruguay), and most countries in the northern region of Africa, Western Asia and Central Asia. Cluster V included most countries close to the Tropic of Capricorn or the Tropic of Cancer, such as Argentina, Australia, Brazil, China, India, Japan, Mauritius, Mexico, South Africa and the USA.

**Figure 4:**
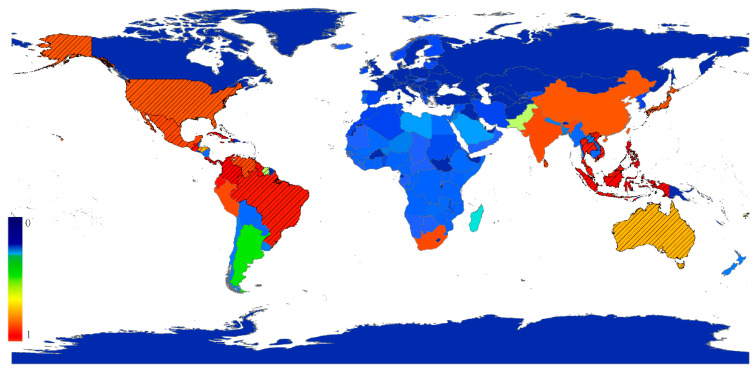
The SOM map and risk distribution of *Furcaspis biformis*. Diagonal lines represent the actual distribution of the species. The value beside the bar is the relative establishment risk score.

Roigé et al., recommend using a ranked list of species as an output of the SOM because of the stability of the ranks, instead of a list of species with a weight value between 0 and 1 which might be easily mistaken for a probability [33]. The top ten ranked species which had high relative establishment risk scores for six countries including Argentina, Australia, China, France, the UK and the USA are listed in Table 1. The risk lists of scale insects for all countries are shown in Table S2 (Supporting Information). For China, *Furcaspis biformis* ranked in 13th place and may be a potential invasive pest in China. As an example, a risk and distribution map of *F. biformis* was generated in order to investigate their potential risk regions (Figure 4).

**Figure 5:**
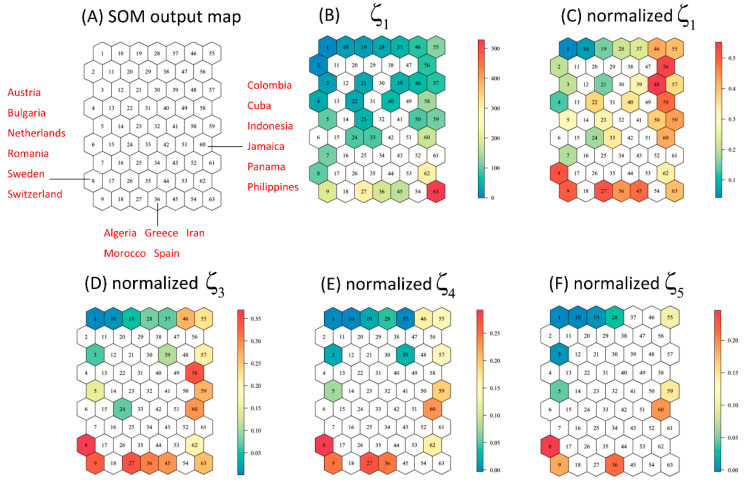
ζ_i_ diversity in SOM map neurons. (**A**) Some regions with high reliability (red text) on the SOM map; (**B**) shows ζ_1_ (average species richness in same neuron); (**C**) shows normalized ζ_2_ (the average number of shared species between any two countries in same neuron); (**D**–**F**) corresponds to normalized ζ_3_, normalized ζ_4_, and normalized ζ_5_.

It is obvious that neurons with low normalized ζ_2–5_ values were mostly clustered in the top left corner of the SOM map, which was consistent with the average species richness (ζ_1_) in each neuron (Figure 5). In neurons 8, 36 and 60, ζ_2_ and ζ_3–5_ are relatively high compared to the other neurons, indicating that low uncertainty was found in these neurons (Table 2). Most neurons of the SOM output had high ζ_2_ and low ζ_3–5_ values, indicating that the risk lists need to be interpreted with caution. The neurons 1, 2, 3, 4, 7, 10, 19, 21 and 24 with low ζ_2–5_ values suggested high uncertainty and should be interpreted with extreme caution (Table 2).

## 4. Discussion

The species number of scale insects on Earth is expected to exceed 10,000 species [34]. Meanwhile, scale insects have strong adaptability when they invade new regions. For example, Pellizzari and Germain reported that alien-scale insects account for about 30% of the total scale insect fauna in Europe [35]. Therefore, it is necessary to assess the risk of invasive scale insects and investigate potential high-risk species in advance. Our analysis correctly predicted China’s most recent invasive pest *Phenacoccus parvus*, which was one of the ten most highly ranked non-established species and strongly associated with the China assemblage. Due to a time lag between recent publication and being included in the database, our data have no occurrence records of *P. parvus* in China. *P. parvus* has been established as an invasive pest in multiple locations in China in recent years [36]. This case indicates that SOM can be a reliable tool for preliminary risk assessments. Not many scale insects are recorded in existing quarantine lists. For example, in the EPPO A1 and A2 lists of pests recommended for regulation as quarantine pests, there are only 5 species of scale insects; in the Catalogue of Quarantine Pests for Import Plants to the People’s Republic of China, 21 species are included; and in the work of Waterhouse and Sands, 22 pest species of scale insects in Australia are described (Table 3) [37]. In contrast, our study provides a ranked list of more than 2000 species for 157 countries. Most species in these regional or national lists have relatively high ranks (top 500) based on the SOM analyses (Table 3). Our results confirm that the SOM method is robust for analyzing a large amount of data and can be an important complement to current consultative practices for predicting invasive risk.

By assessing the similarity of pest assemblages from different countries, China, Japan and the USA were allocated to neuron 63 with medium reliability (Table S1). Geographic units owning similar species assemblages also share similar niches and ecological characteristics [15,38]. The SOM can indicate which countries need the greatest attention from local quarantine departments. As China’s neighbor country, Japan has the most similar scale insect assemblage to China, indicating that any new invasive scale insect from Japan needs Chinese quarantine authorities to be alerted. In 1990, *Matsucoccus matsumurae* (pine bark scale), which is native to Japan, seriously affected 333,000 ha of pines in Northern China, resulting in the loss of 133,000 ha of pines to prevent further invasion [39]. Recently, China and the USA, the two largest economies in the world, were defined as central hubs in the agro-food trade network [40]. It will be a great challenge to prevent the spread of potential invasive scale insect pests between China and the USA, due to their similar species assemblages.

The SOM is a reliable method to rank potential invasive species, but the SOM score is not considered a probability of establishment and a measure of the economic harm of a species on a target region [5]. We have produced the total risk list of scale insects for 157 countries, and suggested using the species ranks instead of risk scores in the list, due to the stability of the ranks as Roigé et al., have shown [33]. Based on the SOM method, a risk list of invasive pests and preliminary risk assessments are able to be established, but an in-depth risk assessment with additional relevant information is needed for assessing the impact of a species on a target region. Singh et al., advised that multi-criteria, such as the existence of particular pathways, host range and survival adaptations, should be used to determine the overall biosecurity risk [15]. SOM analysis is a quantitative tool for analyzing invasive pests, but it does not mean that SOM constitutes an independent and complete pest risk analysis [33]. In the risk list for China, for example, *F. biformis* has a high rank (13). This species has a large range of host plants (11 families) and wide geographic distributions (43 countries) [18]. *F. biformis* is present in China’s neighbors such as Japan, Laos, Thailand and Vietnam (Figure 4), which indicates a potential threat to China. However, *F. biformis* is regarded as a minor pest of orchids [41], because nicotine spray might be effective in killing this scale. Hence *F. biformis* should not be considered a serious invasive pest in China. In contrast, *C. rusci* (rank value = 26) is a serious invasive pest for China, because it has wide range of hosts (with especially serious damage for *Ficus* species) and strong adaption ability in other countries. We suggest that the top species in the risk list (Table S2) should gain more attention from quarantine authorities, though it is hard to define a strict threshold value of species rank. Further analyses are required for assessing the degree of damage and possibility of occurrence imposed by respective pest species.

Predicting the relative establishment risk scores of a large number of species is a significant advantage of the SOM [5,14]. Meanwhile, a low error rate (averaging 8.54%) and high stability of the risk list have been found in SOM analysis [14]. Following the method of Roigé et al., our results show that nine neurons obtain high uncertainty, indicating the risk lists from these neurons need to be interpreted with extreme caution [12]. Generally, the regions with very low species counts or rare species obtained low ζi values [12]. It was consistent with our results that high uncertainty mainly occurs in most countries in Africa with very low species counts. Although ScaleNet, containing a large number of scale insect literature (23,600 references), provides as complete as possible country-level distribution data of scale insects [18], data imbalance is inevitable for some areas. For scale insects, parts of the southern hemisphere and palaeotropics may lack sufficient investigation [24]. Our results (Figure 1) also show that species numbers of many African countries are very low, indicating more investigation may be required in the future. However, the low species number might simply reflect less collecting effort. It is also possible that the distribution patterns noted here are related to host-plant preferences [42]. Even for many polyphagous scale insects, the same situation may also occur. For example, *Ceroplastes rubens* is a well-known polyphagous pest (host families: 79; host genera: 168) [18] with a worldwide distribution; however, it has been rarely collected in Africa and would appear to be restricted to the east coast (Kenya, Tanzania and South Africa) [42]. Numerous factors (host-plant, climate, trade routes, plant or produce importations, etc.) can influence the distribution of phytophagous insect pest species at any geographical location. The presence of suitable host-plants and climate is very crucial for determining the presence of scale insects in some places. Additionally, the number of records for a nation and the cluster to which it is assigned is not easily correlated, and the presence (1) or absence (0) of information on each species should be more important for clustering. For example, the countries of Cluster II include France (399), Italy (412), Turkey (350), Egypt (195) and Morocco (134). The countries of Cluster V include China (1129), Japan (480), Argentina (295) and Mauritius (139). In general, geographically close areas with a similar diversity were grouped into the same cluster. Hence, the SOM shows the advantage of avoiding simple clustering errors based on only species number.

## 5. Conclusions

The present paper provides a preliminary assessment of the establishment risk of scale insects at a global scale. Species with risk ranks were identified for each of the 157 countries (Table S2). Our results contribute to a better understanding of the global distribution of scale insects and help quarantine departments develop appropriate management strategies for a timely interception of invasive scale insects.

## Figures and Tables

**Table 1:** Top ten ranked scale insect species for six countries based on relative establishment risk score (for full list see Table S1). These species are currently absent in these countries.

Rank	China	USA	UK
1	*Uhleria araucariae*	*Nipaecoccus viridis*	*Physokermes piceae*
2	*Odonaspis ruthae*	*Icerya seychellarum*	*Phenacoccus piceae*
3	*Odonaspis saccharicaulis*	*Planococcus minor*	*Sphaerolecanium prunastri*
4	*Ferrisia malvastra*	*Pseudococcus cryptus*	*Diaspidiotus gigas*
5	*Dactylopius ceylonicus*	*Pulvinaria polygonata*	*Coccura comari*
6	*Dactylopius opuntiae*	*Pseudaulacaspis eugeniae*	*Ceroputo pilosellae*
7	*Pulvinaria urbicola*	*Paralecanium expansum*	*Eulecanium franconicum*
8	*Diaspidiotus ancylus*	*Fiorinia turpiniae*	*Lepidosaphes conchiformis*
9	*Phenacoccus parvus*	*Icerya aegyptiaca*	*Heliococcus bohemicus*
10	*Furchadaspis zamiae*	*Aulacaspis madiunensis*	*Acanthococcus aceris*
**Rank**	**Australia**	**France**	**Argentina**
1	*Russellaspis pustulans*	*Oceanaspidiotus spinosus*	*Chrysomphalus aonidum*
2	*Hemiberlesia cyanophylli*	*Chrysomphalus aonidum*	*Ceroplastes floridensis*
3	*Parlatoria cinerea*	*Parlatoria parlatoriae*	*Aulacaspis tubercularis*
4	*Oceanaspidiotus spinosus*	*Anophococcus formicicola*	*Aspidiotus destructor*
5	*Mycetaspis personata*	*Peliococcus turanicus*	*Pinnaspis strachani*
6	*Protopulvinaria pyriformis*	*Lepidosaphes granati*	*Pulvinaria psidii*
7	*Aonidomytilus albus*	*Nipaecoccus nipae*	*Coccus longulus*
8	*Planococcus ficus*	*Peliococcopsis priesneri*	*Ceroplastes sinensis*
9	*Kilifia acuminata*	*Physokermes hemicryphus*	*Coccus viridis*
10	*Nipaecoccus nipae*	*Asterodiaspis minor*	*Russellaspis pustulans*

**Table 2:** The neurons with low and high uncertainties corresponding to normalized ζ_2–5_ values.

Uncertainty	Neuron	ζ_2_	ζ_3_	ζ_4_	ζ_5_	Asia	Africa	North America	South America	Europe	Oceania
low	8	0.52	0.37	0.29	0.24	0	0	0	0	6	0
low	60	0.43	0.29	0.23	0.19	2	0	3	1	0	0
low	36	0.45	0.31	0.25	0.21	1	2	0	0	2	0
high	1	0.05	0.00	0.00	0.00	2	11	0	0	3	1
high	2	0.20	NA	NA	NA	0	0	0	0	2	0
high	3	0.20	0.06	0.02	0.00	1	0	0	0	6	0
high	4	0.13	NA	NA	NA	1	0	0	0	1	0
high	7	0.19	NA	NA	NA	1	0	0	0	1	0
high	10	0.06	0.01	0.00	0.00	3	1	1	0	0	0
high	19	0.15	0.05	0.02	0.01	2	4	1	1	0	0
high	21	0.15	NA	NA	NA	1	0	0	1	0	0
high	24	0.18	0.07	NA	NA	2	0	0	0	1	0

**Table 3:** The SOM ranks of scale insects included in the national quarantine lists of China, Australia and France, with a and p representing absence and presence in ScaleNet dataset.

China ^†^		Australia ^‡^		France ^§^	
Species	Rank	Species	Rank	Species	Ranks
*Ischnaspis longirostris*	22p	*Saissetia oleae*	2p	*Comstockaspis perniciosa*	60p
*Selenaspidus articulatus*	40p	*Coccus hesperidum*	5p	*Maconellicoccus hirsutus*	76p
*Lepidosaphes ulmi*	49p	*Parasaissetia nigra*	6p	*Lopholeucaspis japonica*	120p
*Phenacoccus solenopsis*	63p	*Saissetia coffeae*	7p	*Lepidosaphes ussuriensis*	405a
*Lepidosaphes tokionis*	68p	*Lepidosaphes gloverii*	16p		
*Ceroplastes stellifer*	108p	*Lepidosaphes beckii*	17p		
*Planococcus minor*	112p	*Aonidiella aurantii*	22p		
*Dysmicoccus neobrevipes*	143a	*Planococcus citri*	24p		
*Planococcus lilacius*	154p	*Chrysomphalus aonidum*	29p		
*Ceroplastes rusci*	171a	*Coccus longulus*	41p		
*Epidiaspis leperii*	177p	*Diaspis bromeliae*	44p		
*Aonidiella comperei*	208p	*Ceroplastes floridensis*	46p		
*Carulaspis juniperi*	240a	*Unaspis citri*	47p		
*Hemiberlesia pitysophila*	332p	*Pseudococcus viburni*	59p		
*Eulecanium gigantea*	354p	*Aonidiella orientalis*	64p		
*Dysmicoccus grassi*	438a	*Ceroplastes ceriferus*	69p		
*Lepidosaphes tapleyi*	455p	*Aonidiella citrina*	75p		
*Chionaspis pinifoliae*	491a	*Coccus viridis*	79p		
*Mercetaspis halli*	688a	*Ceroplastes rubens*	85p		
*Parlatoria crypta*	1238a	*Ceroplastes sinensis*	137p		
*Phenacoccus manihoti*	1458a	*Coccus pseudomagnoliarum*	196p		

^†^: Catalogue of Quarantine Pests for Import Plants to the People’s Republic of China (2017). ^‡^: Book of Waterhouse and Sands (2001). ^§^: EPPO A1 and A2 lists of pests recommended for regulation as quarantine pests.

## Data Availability

All the data generated in this work were provided in the article.

## Supplementary Materials

The following supporting information can be downloaded at: https://www.mdpi.com/article/10.3390/insects14070572/s1, Table S1. The original data file of 157 × 2486 matrix including 157 vectors (countries) each with 2486 elements (species). Table S2. SOM risk list of potential invasive scale insect species for 157 countries (all countries are assigned to 63 neurons).
